# Trends and determinants of acute tocolysis implementation in Japan, 2012–2023: An 11-year nationwide retrospective cohort study

**DOI:** 10.1371/journal.pone.0351293

**Published:** 2026-06-22

**Authors:** Mikayo Toba, Rie Oi, Mutsuko Moriwaki, Masayuki Kakehashi, Ayako Fudono, Kiyohide Fushimi, Naoyuki Miyasaka

**Affiliations:** 1 Quality Management Center, Institute of Science Tokyo, Tokyo, Japan; 2 Obstetrics & Gynecology, Tokyo Metropolitan Ohtsuka Hospital, Tokyo, Japan; 3 Graduate School of Biomedical and Health Sciences, Hiroshima University, Hiroshima, Japan; 4 Department of Perinatal and Women’s Medicine, Institute of Science Tokyo Hospital, Tokyo, Japan; 5 Department of Health Policy and Informatics, Institute of Science Tokyo, Tokyo, Japan; 6 Department of Comprehensive Reproductive Medicine, Institute of Science Tokyo, Tokyo, Japan; Baylor College of Medicine, UNITED STATES OF AMERICA

## Abstract

**Background:**

International guidelines, such as those from the WHO, recommend limiting the duration of tocolysis to up to 48 hours in order to provide a window for antenatal corticosteroid administration and maternal transfer. However, reports from multiple countries indicate that tocolysis is being performed for 48 hours or longer in actual clinical settings. This study used nationwide data in Japan over an 11-year period to investigate changes in tocolytic protocol and identify factors associated with tocolysis within 48 hours.

**Methods:**

Using the Diagnosis Procedure Combination database, we analyzed data from 156,356 pregnant women who received ritodrine infusion for preterm labor between April 2012 and March 2023. To accommodate day-based administrative data, we defined acute tocolysis (AT) as ≤2 and maintenance tocolysis (MT) as ≥4 days of treatment. Annual trends in AT utilization rates were examined, and logistic regression analysis was performed to identify factors associated with AT, including institutional characteristics, regional differences, and obstetric complications.

**Results:**

The rate of AT utilization increased from 15.73% to 23.74% over the study period, as did variation among hospitals. University hospitals (adjusted odds ratio [aOR] = 1.16), perinatal centers (aOR = 1.12), preterm premature rupture of membranes (aOR = 3.18), and pregnancy-induced hypertension (aOR = 1.52) were associated with AT, while concomitant use of magnesium sulfate hydrate (aOR = 0.91), multiple fetuses (aOR = 0.64), and placenta previa (aOR = 0.67) were negatively associated with AT utilization.

**Conclusions:**

While AT utilization has increased over time, significant disparities between facilities remain. These findings suggest that the choice of tocolytic protocol is not determined by evidence alone, but is complexly influenced by institutional roles, regional healthcare systems, and specific maternal complications. This study highlights the importance of understanding these multifaceted factors to optimize treatment strategies that balance international standards with the practicalities of individualized patient care.

## Introduction

In 2020, an estimated 13.4 million preterm births were reported worldwide, affecting approximately 1 in 10 newborns [1]. Preterm birth is the leading cause of neonatal and under-five mortality [2,3] and increases the risk of severe childhood illnesses [4,5]. In developed countries, however, the increase in high-risk pregnancies due to maternal aging and assisted reproductive technologies has led to more cases of spontaneous preterm births and medically indicated preterm births, in which early delivery or cesarean section is planned for medical reasons [6–8]. To address this issue, in 2023, the World Health Organization (WHO) recommended the appropriate use of tocolytic agents and the administration of antenatal corticosteroids [9,10]. To reduce maternal adverse events, these recommendations state that the use of tocolytics be limited to a maximum of 48 hours to allow time for antenatal corticosteroid administration and maternal transport to a tertiary perinatal center [11]. Conversely, low compliance with these recommendations and guidelines has been reported in many countries, and in actual clinical practice, tocolytic drug administration continues for more than 48 h [12–14].

Since the 1980s, betamimetics have been widely used as tocolytic agents worldwide. However, in 2013, the European Medicines Agency concluded that high doses of short-acting betamimetics pose a significant risk of severe cardiovascular side effects for both mother and fetus. Consequently, the agency withdrew approval for oral and suppository formulations and restricted the use of injectable formulations to a maximum of 48 h [15].

In 2020, the preterm birth rate in Japan was 5.48%, and the perinatal mortality and neonatal mortality rates were 3.2 and 0.8 per 1,000 live births, respectively, which are the lowest worldwide, indicating that perinatal healthcare in Japan is at the highest global standard [16–18]. On the other hand, tocolytics approved in Japan are limited to betamimetic agents (primarily ritodrine hydrochloride; hereafter referred to as ritodrine) and magnesium sulfate. Unlike international guidelines, the Japanese package inserts for these drugs do not specify a maximum duration for administration; moreover, the Japan Society of Obstetrics and Gynecology (JSOG) guidelines do not strictly prohibit their use beyond 48 hours [19] (S1 Table). Consequently, ritodrine remains the first-line agent, and the traditional clinical practice of maintenance tocolysis (MT), which involves the administration of tocolytics for ≥48 hours, has been widely maintained in Japan [20]. This traditional clinical practice, which favors MT, opposes international evidence-based recommendations such as those from the WHO, which suggest that tocolysis beyond 48 hours provides no significant additional benefit for neonatal outcomes. Following global trends, ritodrine injections decreased by 42.68% in 2022 compared to 2014 [21]. However, the specific clinical drivers behind this decline remain unclear; moreover, it is unknown whether this decline represents a systematic shift toward evidence-based acute tocolysis (AT) [22,23].

Given the global shift toward evidence-based practice, it is crucial to determine whether Japan’s unique clinical landscape is transitioning from traditional long-term MT to AT per international standards. Confirmation of this trend would represent a positive evolution in Japanese perinatal care—one that optimizes maternal safety without compromising its world-leading neonatal outcomes. Accordingly, the aim of this study was to clarify the current status of tocolytic practice in Japan and its evolution over the past 11 years. In addition, the institutional and clinical factors driving the transition in tocolytic practice were identified. By analyzing these determinants, we sought to provide an empirical basis for balancing international evidence with the practical realities of individualized perinatal care in Japan.

## Materials and methods

### Study design and setting

This retrospective cohort study used the Diagnosis Procedure Combination (DPC) database, a national administrative claim database for acutecare inpatients in Japan. This database does not include data from private clinics, as they operate under a different reimbursement system. As of 2023, the DPC database covered 1,761 facilities and 483,459 beds, representing approximately 55% of all general hospital beds in Japan [24,25]. Although this database does not cover all medical institutions, it encompasses nearly all academic hospitals and regional perinatal centers. Because intensive tocolytic management for preterm labor—specifically intravenous administration—is primarily centralized in these advanced-care facilities, this dataset strongly reflects the nationwide clinical trends in Japan. For each patient, the database provides comprehensive data, including demographics, diagnoses coded by the International Classification of Diseases 10th revision (ICD-10), daily records of medical procedures and medications, admission/discharge dates, and clinical outcomes [25]. Specifically for the perinatal field, it captures essential information such as gestational age at admission and details of delivery during hospitalization. The reliability and validity of the DPC database for clinical epidemiological research have been well-established in previous studies [26–28].

### Study population

The study participants were pregnant women aged 22 ⁺ 0–36 ⁺ 6 weeks of gestational age who were hospitalized between April 1, 2012, and March 31, 2023, and received ritodrine hydrochloride injection. In Japan, tocolytic therapy is indicated for not only “preterm labor” (defined as regular painful contractions with cervical changes) but also “threatened preterm labor,” which may involve uterine contractions alone regardless of the cervical status. As this study used a nationwide administrative database, the study population encompassed the full clinical spectrum of conditions for which intravenous ritodrine was clinically indicated according to Japanese practice standards.

Patients with unknown gestational age and those transferred from another hospital were excluded, as their prior treatment status could not be ascertained. For research purposes, the dataset was first accessed on 05/02/2025.

Because the DPC database records medication data on a per-day basis, precise hourly durations cannot be determined. To ensure a clear distinction between short-term and prolonged therapy, we categorized patients into two groups: the AT group, including those receiving ritodrine for ≤2 days, and the MT group, including those receiving ritodrine for ≥4 days. Patients with a 3-day duration were excluded from the analysis to avoid potential misclassification, as this group likely contained a mix of both acute and maintenance treatment profiles.

### Variables

The variables analyzed in this study included maternal age; the Charlson Comorbidity Index [29,30]; obstetric diseases—such as preterm premature rupture of membranes (pPROM), hypertensive disorder of pregnancy (HDP), placenta previa, and cervical insufficiency; pregnancy status—specifically gestational age and singleton pregnancy—along with academic hospital status, perinatal medical center designation, hospital region, and fiscal year at admission. Academic hospitals were identified as DPC Group 1 (university hospitals). Perinatal medical centers were defined as facilities that claimed the comprehensive perinatal intensive care unit management fee. The hospitals were categorized into six regions based on geographical location: Hokkaido-Tohoku, Kanto, Chubu, Kansai, Chugoku-Shikoku, and Kyushu-Okinawa. Because these institutional categories may overlap, we assessed the variance inflation factor (VIF) to detect potential multicollinearity among variables in the multivariable models. All VIF values were within acceptable limits, confirming the independent contribution of each variable.

### Statistical analysis

First, we measured the AT utilization rate on both a patient-by-patient and a hospital-by-hospital basis. We analyzed annual trends in AT utilization rates using the Jonckheere-Terpstra test, to evaluate the presence of monotonic trends over time. Next, patients were divided into groups according to the method of tocolytic agent administration, after which patient and hospital characteristics were summarized using appropriate descriptive statistics. Continuous variables are expressed as means with standard deviations (SD) and were compared between groups using the Mann–Whitney U test. Categorical variables are presented as frequencies with percentages and were compared using the chi-square test. Logistic regression analysis (forced entry method) was then performed with AT utilization as the dependent variable. Results are presented as adjusted odds ratios (aOR) with 95% confidence intervals (CI). SPSS version 30 (IBM, Chicago, Illinois, USA) was used for all analyses, and the significance level was set at 5% (two-tailed test).

### Ethical considerations

The Institute of Science Tokyo Faculty of Medicine Ethics Review Committee approved this study (approval number: M2000-788; date of approval: December 19, 2023). The committee waived the requirement for informed consent because all personal information was excluded, and de-identified data were provided to the researchers for secondary use. This study was conducted in accordance with the principles of the Declaration of Helsinki.

## Results

### Participants

**Fig 1:**
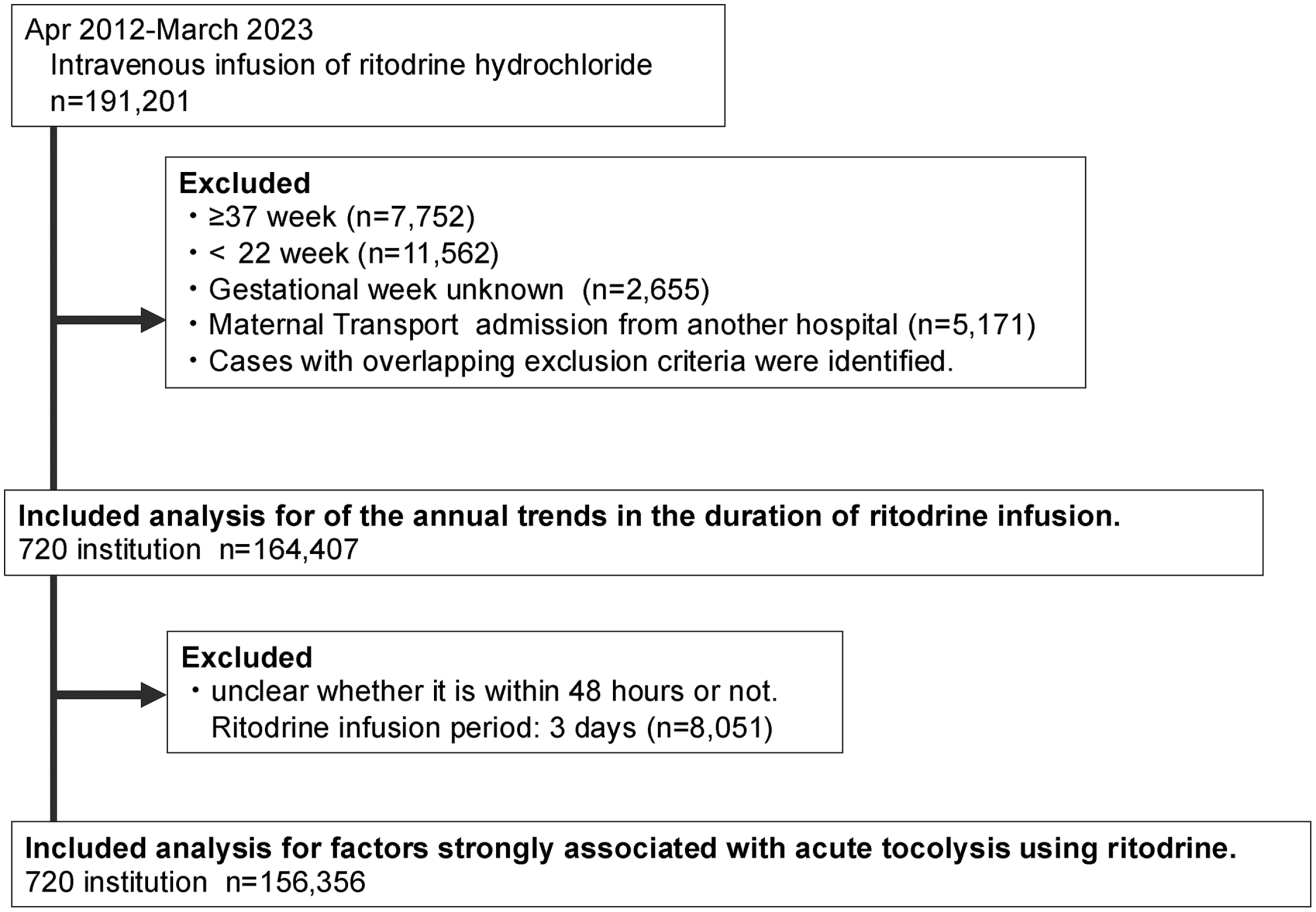
Flowchart of study population. The annual trends by duration of ritodrine administration were based on 164,407 patients from 720 hospitals who were hospitalized and received intravenous ritodrine hydrochloride from April 2012 to March 2023, excluding patients with a pregnancy of 37 weeks or more, less than 22 weeks, unknown gestational age, and those admitted for maternal transfer after discharge. The analysis of factors associated with AT included 156,356 patients, excluding patients with a mixed AT and MT who received ritodrine for exactly 3 days.

During the study period, 191,201 patients were identified who received ritodrine infusion. We first established a population of 164,407 patients from 720 hospitals to analyze annual trends. This cohort excluded those with unknown or ineligible gestational age (<22 or ≥ 37 weeks) and patients transferred from other facilities, ensuring the analysis reflected the full course of inpatient management (Fig 1). For the subsequent analysis of factors associated with tocolytic protocols, we further excluded patients who received ritodrine for exactly 3 days. This was done to minimize misclassification between the two distinct treatment strategies. Consequently, 156,356 patients were included in the multivariable logistic regression analysis to compare the AT and MT.

**Fig 2:**
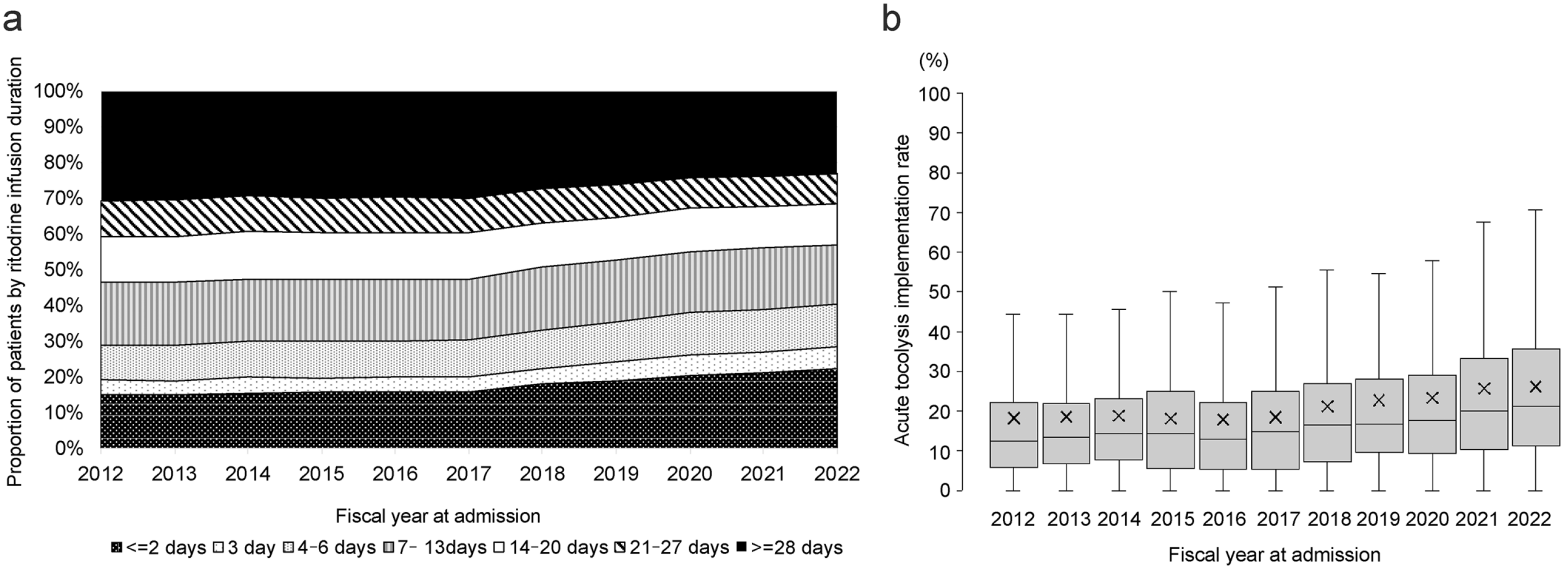
Infusion period of ritodrine hydrochloride. a: Trends in ritodrine infusion duration among all study participants (n = 164,407). Stacked area chart showing the annual proportions of tocolytic treatment durations from 2012 to 2022. The vertical axis represents the percentage of patients, and the horizontal axis denotes the fiscal year. The area for acute tocolysis (AT, ≤ 2 days) demonstrates a significant increasing trend (Jonckheere-Terpstra test: p = 0.036). The utilization rate of AT (≤2 days) showed an increasing trend (Jonckheere-Terpstra test: p = 0.036). b: Annual trends and inter-hospital variation in AT utilization rates (n = 720 hospitals). The box-and-whisker plots illustrate the distribution of AT utilization rates (≤ 2 days) across facilities for each fiscal year. The horizontal line within each box represents the median, while the × symbol indicates the mean utilization rate. The box boundaries indicate the interquartile range (IQR), and the whiskers extend to the minimum and maximum values (excluding outliers). While a significant increasing trend was observed (Jonckheere-Terpstra test: p = 0.016), the widening of the boxes and whiskers reflects the increasing diversity in clinical protocols among institutions.

The tocolysis duration significantly decreased over time (Fig 2a). The Jonckheere–Terpstra test confirmed significant directional shifts at both ends of the duration spectrum. The proportions of patients in the groups receiving ≥ 14 days of tocolysis showed a significant decreasing trend, while those receiving ≤ 2 days significantly increased. In contrast, no significant trends were observed in the intermediate groups with tocolysis durations of 3–13 days (Fig 2a, S2 Table). The AT utilization rate by hospitals also showed a monotonically increasing trend, although the variation between hospitals increased over time (Fig 2b).

Table 1 summarizes the patient characteristics categorized by tocolytic duration. The mean maternal age was similar between the two groups (AT: 31.92 ± 5.48 years; MT: 31.76 ± 5.41 years), although the difference reached statistical significance due to the large sample size. Notably, the mean gestational age at admission was significantly higher in the AT group compared with the MT group (31.89 ± 4.41 weeks vs. 29.02 ± 4.40 weeks, p < 0.001). Regarding obstetric complications, patients with pPROM or HDP were more frequently managed with AT. Conversely, multiple pregnancies, placenta previa, and cervical insufficiency were more prevalent in the MT group. Institutional characteristics also influenced treatment, with academic hospitals and perinatal centers showing a higher tendency toward AT. Additionally, the AT group had a higher rate of antenatal corticosteroid administration (23.90% vs. 21.99%, p < 0.001), while the concomitant use of magnesium sulfate was more frequent in the MT group (23.30% vs. 19.05%, p < 0.001).

**Table 1:** Characteristics of the participants according to ritodrine hydrochloride infusion (N = 156,356).

	Acute tocolysis^a^ N = 29,314		Maintenance tocolysis^b^ N = 127,042		p^c^
**Maternal characteristics of admission**					
Age (years) [mean, SD^d^]	31.92	5.48	31.76	5.41	<0.001
Gestational age (weeks) [mean, SD^d^]	31.89	4.41	29.02	4.40	<0.001
BMI^e^ (kg/m^2^) [mean, SD^d^]	23.66	4.35	23.06	3.72	<0.001
pPROM^f^, n,%	6,607	22.54	10,691	8.42	<0.001
Multiple pregnancy, n,%	2,362	8.06	15,010	11.81	<0.001
Placenta previa, n,%	1,692	5.77	10,285	8.10	<0.001
Cervical insufficiency, n,%	1,172	4.00	8,454	6.65	<0.001
Gestational diabetes mellitus, n,%	1,352	4.61	7,259	5.71	<0.001
Hypertensive disorders of pregnancy, n,%	1,639	5.59	4,982	3.92	<0.001
CCI^g^, ≥ 1, n,%	847	2.89	3,769	2.97	0.481
**Institution**					
Academic hospital, n,%	8,442	28.80	34,042	26.80	<0.001
Perinatal medical center, n,%	11,986	40.89	49,563	39.01	<0.001
**Regions of the hospital**					
Hokkaido-Tohoku, n,%	3,790	12.93	21,578	16.98	<0.001
Kanto, n,%	7,769	26.50	32,546	25.62	
Chubu, n,%	5,477	18.68	26,779	21.08	
Kansai, n,%	5,401	18.42	19,748	15.54	
Chugoku-Shikoku, n,%	3,764	12.84	16,297	12.83	
Kyushu-Okinawa, n,%	3,113	10.62	10,094	7.95	
**Fiscal year**					
2012, n,%	1,774	6.05	9,512	7.49	<0.001
2013, n,%	1,814	6.19	9,825	7.73	
2014, n,%	2,429	8.29	12,567	9.89	
2015, n,%	2,336	7.97	11,868	9.34	
2016, n,%	2,191	7.47	11,290	8.89	
2017, n,%	2,057	7.02	10,545	8.30	
2018, n,%	2,564	8.75	11,047	8.70	
2019, n,%	3,339	11.39	13,387	10.54	
2020, n,%	3,503	11.95	12,661	9.97	
2021, n,%	3,809	12.99	13,104	10.31	
2022, n,%	3,498	11.93	11,236	8.84	
**Preterm labor management**					
ACS^h^, n,%	7,005	23.90	27,934	21.99	<0.001
Magnesium sulfate hydrate, n,%	5,583	19.05	29,600	23.30	<0.001

^a^acute tocolysis: ritodrine hydrochloride infusion for 2 days or less.

^b^maintenance tocolysis: ritodrine hydrochloride infusion for more than 4 days.

^c^continuous variable: Mann-Whitney U test, discrete variable：chi-square test.

^d^standard deviation.

^e^body mass index.

^f^preterm premature rupture of membranes.

^g^Charlson comorbidity index.

^h^antenatal corticosteroid administration.

**Fig 3:**
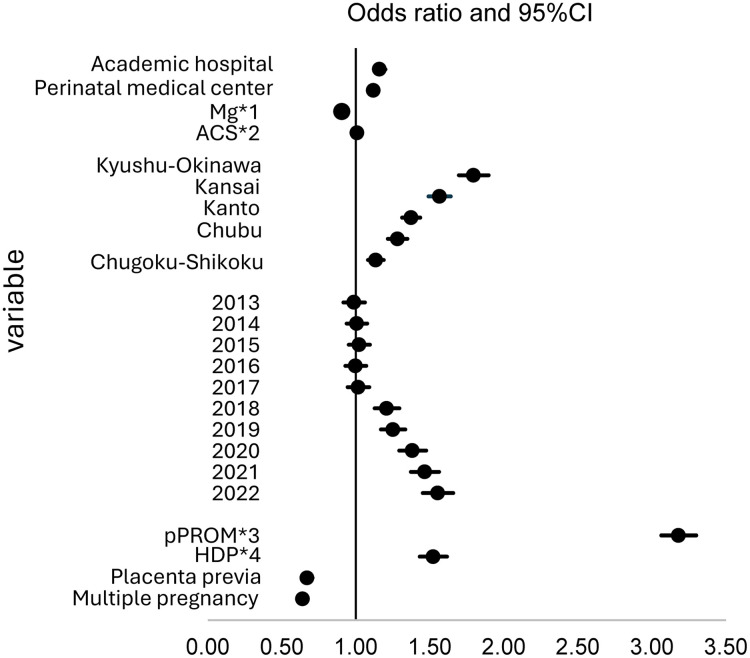
Forest plot of factors associated with the utilization of acute tocolysis. A multivariable logistic regression analysis was performed to identify factors associated with AT (≤ 2 days). The dependent variable was the presence of AT (≤ 2 days). The model was adjusted for the following covariates: preterm labor management (magnesium sulfate use and antenatal corticosteroid administration), healthcare provider factors (academic hospital and perinatal medical center), region (reference: Hokkaido-Tohoku), fiscal year, and maternal obstetric complications (pPROM, HDP, gestational age, placenta previa, multiple pregnancy, and cervical insufficiency). *1: magnesium sulfate, *2: antenatal corticosteroid, *3: preterm premature rupture of membranes, *4: hypertensive disorder of pregnancy.

The results of the multivariable logistic regression analysis are presented in Fig 3 and S3 Table. Institutional factors significantly influenced the choice of protocol; admission to academic hospitals (aOR: 1.16; 95% CI: 1.12–1.20) and perinatal centers (aOR: 1.12; 95% CI: 1.08–1.15) were positively associated with AT use. We also observed a distinct regional gradient, with the Kyushu-Okinawa region showing the strongest association with AT (aOR: 1.79; 95% CI: 1.70–1.90) compared to the reference region. Furthermore, the likelihood of AT utilization increased progressively over time, particularly from 2018 onward, reaching an aOR of 1.55 (95% CI: 1.45–1.66) in 2022 compared to 2012 (all p < 0.001). Regarding maternal obstetric complications, pPROM (aOR: 3.18; 95% CI: 3.06–3.30) and HDP (aOR: 1.52; 95% CI: 1.43–1.62) were associated with AT. In contrast, multiple pregnancies (aOR: 0.64; 95% CI: 0.61–0.67) and placenta previa (aOR: 0.67; 95% CI: 0.63–0.71) were associated with MT. For every one-week increase in gestational age at admission, the odds of receiving AT increased by 1.13 times (95% CI: 1.12–1.14).

## Discussion

To our knowledge, this nationwide retrospective cohort study is the first to report an 11-year longitudinal trend in the transition toward AT in Japan. Analyzing a large-scale dataset of 156,356 pregnant women, we identified key obstetric and institutional factors influencing the choice of AT. Our findings revealed a steady increase in AT utilization over the past decade; however, it remains a minority practice compared with the use of long-term regimens. Furthermore, the results highlight a widening disparity in clinical practices among hospitals.

### Trends in AT adoption

International guidelines generally recommend AT because there is no robust evidence that tocolysis beyond 48 hours improves neonatal outcomes [7,11].Conversely, adherence to guidelines is reported to be low, and in actual clinical practice, MT after AT and the administration of tocolytic agents for threatened preterm labor are still performed [12–14,31,32]. Rousseau et al. identified barriers to guideline adherence, including physicians over 50 years of age, those working in non-university hospitals, and those fearing change [31]. Additionally, Lee et al. stated that patient opinion plays a major role in obstetricians’ decision-making, and that maintenance medications may be prescribed based on the patient’s request [32].

In Japan, ritodrine is the first-line drug for preterm labor, and its use for MT aligns with both domestic guidelines and the package insert [19]. The JSOG guidelines, revised every three years since 2008, have played a key role in maintaining Japan’s low preterm birth rate, even as maternal aging and the use of assisted reproductive technologies have increased [17]. The JSOG guidelines first introduced the international trend of AT utilization in 2014 by citing the European Medicines Agency’s 48-hour restriction and the Food and Drug Administration’s warnings. While the JSOG did not formally restrict the duration of tocolysis to 48 hours—maintaining that the decision should be based on clinical context and maternal safety—the repeated inclusion of these international perspectives in subsequent revisions (2017, 2020, and 2023) likely increased awareness among Japanese clinicians. This likely contributed to the shift toward AT, particularly in academic centers where physicians are more exposed to international evidence. The rate of ritodrine injection use in Japan decreased by 15.3% from 2017 to 2018 and increased to 47.4% by 2022 [21]. Multivariate logistic regression analysis suggested that the association with AT has strengthened over time since 2018, likely due to the influence of guideline descriptions such as this. Furthermore, patients treated at academic hospitals and perinatal centers were strongly associated with AT. Many of the committee members who create the guidelines are doctors employed at such facilities, and it is speculated that their frequent exposure to diverse information and publications from Japan and abroad influences their choice of AT. This finding is consistent with the results of Rousseau, et al. [31].

Interestingly, ACS administration was not a significant predictor of AT in our study results; the administration rates were 23.90% in the AT group and 21.99% in the MT group. This lack of association reflects the unique clinical landscape in Japan, where tocolytic duration and ACS administration are often handled as independent clinical decisions. Unlike in many Western countries, where tocolysis is primarily indicated as a 48-hour bridge for ACS, Japanese clinical practice has historically focused on long-term pregnancy prolongation. Consequently, the choice of AT in Japan may be driven more by factors such as the physician’s adherence to global evidence-based trends or the severity of symptoms, rather than being specifically coordinated with the ACS administration window.

### Regional and institutional variations in tocolytic practice

This study showed that regional factors are associated with AT use. Previous research has reported that differences in the provision of perinatal medical care between urban and rural areas, as well as availability of obstetricians and gynecologists, can influence medical decision-making and care for mothers and children [33,34]. It is hypothesized that in areas with low population density and vast land area, factors such as long travel distances for obstetric checkups and births, difficulty in transporting the mother due to snow in winter, and availability of obstetricians and pediatricians can influence the choice of MT [35,36]. These findings indicate the importance of selecting the most appropriate treatment for mothers and children based on local healthcare infrastructure and regional conditions.

### Maternal and obstetric conditions influencing tocolysis

The utilization rate of AT has increased over the past 11 years; however, the preterm birth rate in Japan has remained stable at 5.48%–5.75% for those under 37 weeks of gestation and 0.25%–0.26% for those under 28 weeks of gestation [16]. It is noteworthy that while the utilization of long-term tocolysis decreased and that of AT increased over the 11-year period, the preterm birth rate in Japan remained stable, being significantly lower than that in the United States, which ranges from 9.7% to 10.4% [36]. Although the contribution of tocolysis beyond 48 hours to a further reduction in preterm birth rates remains controversial, the stability of Japan’s preterm birth rate, despite a decrease in long-term tocolysis, suggests that the shift toward shorter-term management has not compromised overall neonatal outcomes.

Enengl et al. identified placenta previa as a specific indication for MT [13]. Similarly, our findings indicate that placenta previa is more strongly associated with MT. In contrast, conditions such as pPROM and severe HDP, which require careful management due to the potential adverse effects of prolonged gestation on maternal and fetal outcomes, are more strongly associated with AT. In addition, the early gestational age at admission tends to be more closely linked to MT. These findings suggest that the adoption of AT is not uniform across facilities and that treatment options are often selected based on the maternal condition and clinical context.

### Importance of shared decision-making in tocolysis

Major international guidelines, including those from the WHO, ACOG, and NICE, emphasize that tocolytic therapy must involve shared decision-making (SDM). This process requires providing pregnant women and their families with comprehensive information regarding the risks and benefits of both AT and MT, potential neonatal outcomes, and the limitations of survival at various gestational ages [7,11]. While the present study—relying on administrative data—could not directly evaluate the quality of these counseling processes, our findings highlight a significant gap: there is still a lack of evidence-based frameworks to guide SDM that account for regional healthcare infrastructure, financial resources, and geographical constraints. Future research should prioritize addressing these factors to facilitate more personalized and informed decision-making in tocolytic management [9].

### Strengths and limitations of the study

A major strength of this study is its longitudinal design, covering an 11-year period across 720 hospitals, which ensures high representativeness of the clinical landscape in Japan. Furthermore, our methodology—using administrative claims data to analyze long-term trends and factors influencing clinical practice—offers a scalable model for similar investigations in other countries.

However, several limitations must be acknowledged. First, the retrospective nature of this cohort study introduces the potential for “confounding by indication”; patients receiving different treatment durations likely varied in clinical severity or other unmeasured factors that influenced the initial choice of therapy. Second, the use of the DPC database limits access to granular clinical details, such as cervical length, fetal fibronectin levels, or the specific qualitative rationale behind a physician’s decision. Third, the DPC exclusively covers acute care hospitals and excludes private clinics. While some low-risk patients may receive initial treatment at clinics, our study focuses on facilities where critical decisions such as transition to AT and corticosteroid administration are primarily made. Thus, our findings provide a crucial benchmark for high-risk perinatal care in Japan. Finally, the generalizability of our results may be limited; ritodrine is not available in the United States, and the Japanese definition of “threatened preterm labor” is broader than that per international standards. Therefore, while our study findings reliably reflect Japanese clinical practice, the comparative effectiveness of these trends with regard to long-term neonatal outcomes requires further investigation.

### Implications for clinical practice and future research

The findings of this study have important implications for clinical practice and education. By identifying factors that influence the use of AT, this study provides evidence for obstetricians to tailor treatment choices to guidelines and patient conditions. These results may also support shared decision-making. Furthermore, these findings may raise awareness among trainees about unresolved challenges in routinely performed tocolysis. Future prospective studies should compare maternal and neonatal outcomes of long-term versus short-term tocolysis in Japan and include economic evaluations of prolonged therapy. Therefore, to maintain and improve the quality of maternal and child health, further research is needed to establish protocols and evaluate available tocolytic drugs, taking into consideration work style reforms for doctors, shortages of obstetricians and pediatricians, and geographical issues.

## Conclusions

Although the overall rate of AT utilization has increased, compared to MT, it remains a minority practice. There are significant differences in utilization across facilities, and these gaps are widening. AT use tended to be influenced by factors such as hospital functions, obstetric complications, and regional characteristics. Further studies should explore the optimal tocolytic therapy strategy that minimizes maternal and fetal adverse events while improving neonatal outcomes. Simultaneously, pregnant women and their families as well as healthcare professionals should be made aware of the ongoing debate regarding the optimal duration of tocolysis. Currently, robust evidence regarding an improvement in neonatal outcomes with tocolysis extending beyond 48 hours is lacking. These uncertainties underscore the need for a more evidence-based, individualized approach in clinical practice.

## Supporting information

S1 Table Summary of international and domestic guidelines regarding the duration of tocolytic administration. (DOCX)

S2 Table Annual trends in the duration of ritodrine hydrochloride administration. (DOCX)

S3 Table Multivariate logistic regression analysis of factors associated with acute tocolysis using ritodrine hydrochloride infusion. (DOCX)
