# Supplementary material for: Trends and determinants of acute tocolysis implementation in Japan, 2012–2023: An 11-year nationwide retrospective cohort study
Source: PLoS One. 2026 Jun 22;21(6):e0351293. doi: 10.1371/journal.pone.0351293 (PMC13286165; doi:10.1371/journal.pone.0351293)
Supplement: S1 Table — (DOCX) [file pone.0351293.s001.docx]

**S1 Table.** Summary of international and domestic guidelines regarding the duration of tocolytic administration

| **Organization** | **First-line Tocolytics** | **Recommended Duration** |
| --- | --- | --- |
| **JSOG** (Japan) | Ritodrine, Magnesium sulfate | No strict limit |
| **WHO** (International) | Nifedipine | Up to 48 hours |
| **ACOG** (USA) | Nifedipine, Indomethacin | Up to 48 hours |
| **RCOG/NICE**（UK） | Nifedipine | Up to 48 hours |

JSOG: Japan Society of Obstetrics and Gynecology

WHO: World Health Organization

ACOG: American College of Obstetricians and Gynecologists

RCOG: Royal College of Obstetricians and Gynaecologists

NICE: National Institute for Health and Care Excellence
