# Supplementary material for: Trends and determinants of acute tocolysis implementation in Japan, 2012–2023: An 11-year nationwide retrospective cohort study
Source: PLoS One. 2026 Jun 22;21(6):e0351293. doi: 10.1371/journal.pone.0351293 (PMC13286165; doi:10.1371/journal.pone.0351293)
Supplement: S2 Table — (DOCX) [file pone.0351293.s002.docx]

**S2 Table. Annual trends in the duration of ritodrine hydrochloride administration**

|  | **2012** | **2013** | **2014** | **2015** | **2016** | **2017** | **2018** | **2019** | **2020** | **2021** | **2022** | **P^*1^** |
| --- | --- | --- | --- | --- | --- | --- | --- | --- | --- | --- | --- | --- |
| ≤2 days | 15.08% | 14.94% | 15.49% | 15.79% | 15.51% | 15.59% | 18.03% | 18.94% | 20.43% | 21.25% | 22.26% | 0.029 |
| 3 days | 3.95% | 3.94% | 4.28% | 3.88% | 4.45% | 4.37% | 4.28% | 5.07% | 5.71% | 5.56% | 6.17% | 0.532 |
| 4–6 days | 9.94% | 10.09% | 10.29% | 10.20% | 9.99% | 10.44% | 10.84% | 11.42% | 11.71% | 12.07% | 11.81% | 0.815 |
| 7–13 days | 17.62% | 17.42% | 17.29% | 17.25% | 17.22% | 16.97% | 17.45% | 17.35% | 17.27% | 17.36% | 16.69% | 0.102 |
| 14–20 days | 12.64% | 12.87% | 13.58% | 13.36% | 13.03% | 12.85% | 12.54% | 11.80% | 12.14% | 11.39% | 11.41% | 0.004 |
| 21–27 days | 10.08% | 10.34% | 9.74% | 9.61% | 10.22% | 9.75% | 9.59% | 9.27% | 8.58% | 8.45% | 8.53% | 0.036 |
| ≥28 days | 30.68% | 30.39% | 29.33% | 29.91% | 29.57% | 30.03% | 27.28% | 26.15% | 24.16% | 23.92% | 23.12% | 0.036 |

^*1^Jonckheere–Terpstra
