# Supplementary material for: Trends and determinants of acute tocolysis implementation in Japan, 2012–2023: An 11-year nationwide retrospective cohort study
Source: PLoS One. 2026 Jun 22;21(6):e0351293. doi: 10.1371/journal.pone.0351293 (PMC13286165; doi:10.1371/journal.pone.0351293)
Supplement: S3 Table — (DOCX) [file pone.0351293.s003.docx]

**S3 Table.** Multivariate logistic regression analysis of factors associated with acute tocolysis using ritodrine hydrochloride infusion

|  | Odds ratio | 95% CI^*1^ | | p |
| --- | --- | --- | --- | --- |
|  |  | Lower | Upper |  |
| **Preterm labor management** |  |  |  |  |
| Magnesium sulfate hydrate | 0.91 | 0.87 | 0.94 | <0.001 |
| Antenatal corticosteroids administration | 1.01 | 0.97 | 1.04 | 0.687 |
| **Healthcare provision factors** |  |  |  |  |
| Academic hospital | 1.16 | 1.12 | 1.20 | <0.001 |
| Perinatal medical center | 1.12 | 1.08 | 1.15 | <0.001 |
| Reference region: Hokkaido-Tohoku |  |  |  | <0.001 |
| Kyushu-Okinawa | 1.79 | 1.70 | 1.90 | <0.001 |
| Kansai | 1.56 | 1.49 | 1.64 | <0.001 |
| Kanto | 1.37 | 1.31 | 1.44 | <0.001 |
| Chugoku-Shikoku | 1.28 | 1.22 | 1.35 | <0.001 |
| Chubu | 1.13 | 1.08 | 1.19 | <0.001 |
| Reference fiscal year: 2012 |  |  |  | <0.001 |
| 2013 | 0.99 | 0.92 | 1.06 | 0.713 |
| 2014 | 1.01 | 0.94 | 1.08 | 0.885 |
| 2015 | 1.02 | 0.95 | 1.10 | 0.555 |
| 2016 | 1.00 | 0.93 | 1.07 | 0.942 |
| 2017 | 1.02 | 0.94 | 1.09 | 0.682 |
| 2018 | 1.21 | 1.13 | 1.29 | <0.001 |
| 2019 | 1.25 | 1.17 | 1.33 | <0.001 |
| 2020 | 1.38 | 1.29 | 1.48 | <0.001 |
| 2021 | 1.46 | 1.37 | 1.56 | <0.001 |
| 2022 | 1.55 | 1.45 | 1.66 | <0.001 |
| **Maternal obstetric complications** |  |  |  |  |
| pPROM^*2^ | 3.18 | 3.06 | 3.30 | <0.001 |
| Hypertensive disorders of pregnancy | 1.52 | 1.43 | 1.62 | <0.001 |
| Gestational age | 1.13 | 1.13 | 1.14 | <0.001 |
| Placenta previa | 0.67 | 0.63 | 0.71 | <0.001 |
| Multiple pregnancy | 0.64 | 0.61 | 0.67 | <0.001 |
| Cervical insufficiency | 1.03 | 0.96 | 1.10 | 0.373 |

^*1^Confidence interval

^*2^Preterm premature rupture of membranes
